# Metastatic Infiltrating Lobular Breast Cancer to the Colon Diagnosed Through Routine Bowel Screening in a 67-Year-Old Female

**DOI:** 10.7759/cureus.29279

**Published:** 2022-09-17

**Authors:** Jee Eun Do, Timothy Ganguly, Sean Chang, Devinder Raju

**Affiliations:** 1 Department of Surgery, Lyell McEwin Hospital, Elizabeth Vale, AUS; 2 Department of Pathology, Lyell McEwin Hospital, Elizabeth Vale, AUS

**Keywords:** colorectal metastasis, fobt, invasive lobular breast carcinoma, infiltrating lobular carcinoma, infiltrating ductal carcinoma, breast cancer, breast cancer metastasis

## Abstract

Breast cancer most commonly metastasizes to the bone, lung, liver, and brain. The colon is an uncommon site for metastases and its symptoms are variable. A 67-year-old female with a history of breast cancer was referred for colonoscopy following a positive fecal occult blood test (FOBT); there were no discrete lesions concerning for primary colonic cancers or metastasis; however, a random biopsy revealed metastatic breast cancer. The possibility of colonic metastases must be considered when assessing positive FOBT in a patient with previous breast cancer.

## Introduction

Breast cancer is the second most common cancer diagnosed in Australia and the most common cancer in women, with the estimated incidence in 2022 being over 20,600 cases [1]. Common sites for distant metastases are the bone (59.9%), lung (47.8%), liver (40.9%), and brain (38.8%) [2], and metastases to the gastrointestinal tract, especially the colon, are uncommon. The diagnosis of colonic metastasis is difficult because of its rarity, and there is also a long disease-free interval between the diagnosis of breast cancer to the diagnosis of gastrointestinal metastases [3]. We report a case of an incidental finding of colonic metastasis through a screening fecal occult blood test, to alert surgeons that the possibility of metastases should be considered when assessing a patient for a positive result, in those with a history of breast cancer.

## Case presentation

A 67-year-old female was diagnosed with pT1a pN1 M0 infiltrating lobular carcinoma of the right breast in 2013. She underwent a bilateral mastectomy and axillary clearance due to personal preference, followed by adjuvant chemotherapy with 5-fluorouracil, epirubicin, and cyclophosphamide. Following this, she had been treated with palbociclib after failed treatment with an aromatase inhibitor and ribociclib due to intolerance.

**Figure 1:**
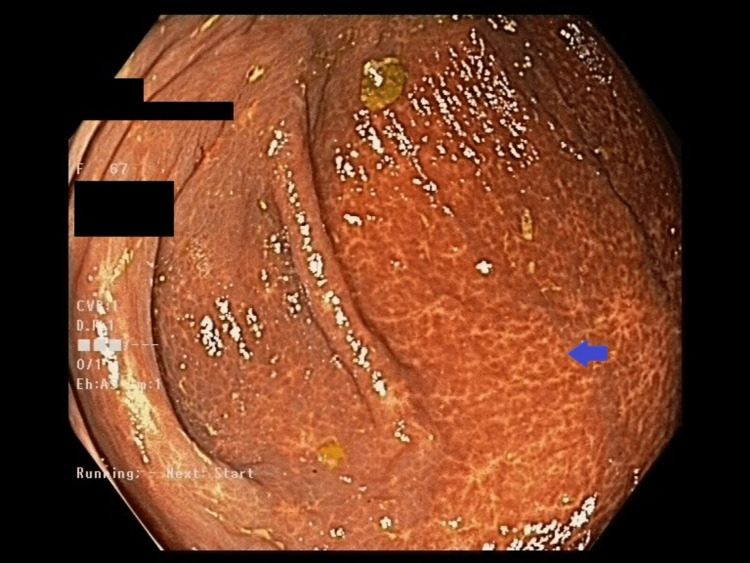
Macroscopic appearance of the colon during colonoscopy Colonoscopy showing melanosis coli. No lesions were found.

**Figure 2:**
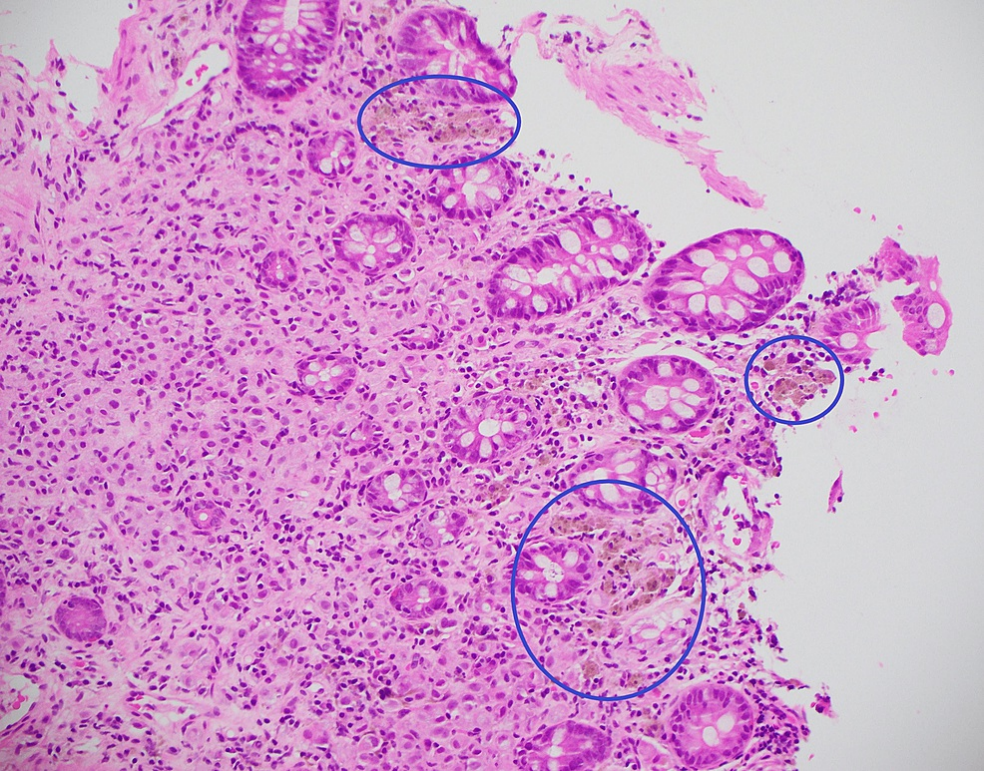
Random biopsy of the ascending colon The ascending colon biopsy, with hematoxylin and eosin stain, showing clusters of atypical cells in the lamina propria, consistent with metastatic lobular breast carcinoma.

**Figure 3:**
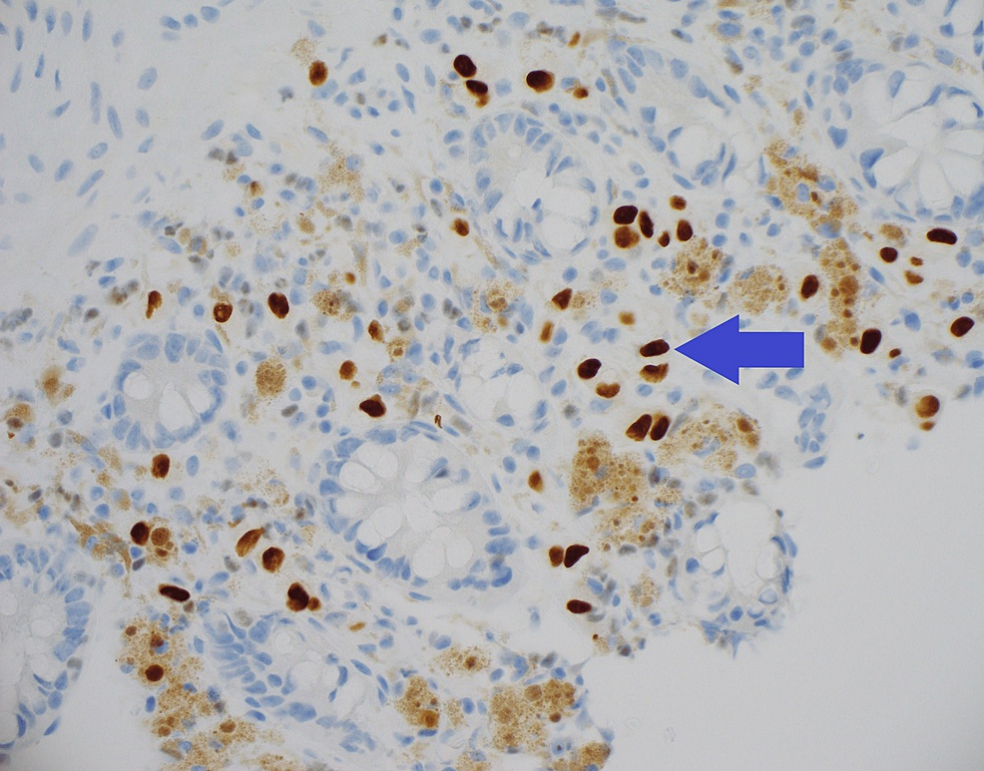
Random biopsy of the ascending colon with the immunoperoxidase GATA3 stain Immunoperoxidase stain for GATA3 is positive in the tumor cells, confirming the presence of lobular breast cancers within the bowel wall.

Seven years after the diagnosis of breast cancer (BC), she was referred for a colonoscopy when a screening fecal occult blood test (FOBT) returned a positive result. She had been up to date with her bowel screening and had a regular follow-up, with all surveillance scans being negative for distant metastases and local recurrence. The colonoscopy revealed melanosis coli due to previous stimulant laxative use, however, no other abnormalities or lesions were seen (Figure 1). Random biopsies were taken, which unexpectedly revealed infiltration of the lamina propria by atypical cells (Figure 2) positive for GATA-3, consistent with metastatic BC (Figure 3) [4]. Further investigation with computer tomography (CT) and a whole-body bone scan did not show any other distant metastases. The patient decided not to pursue any active treatment and was referred to the palliative care team.

## Discussion

BC is subcategorized into ductal and lobular carcinoma, with infiltrating ductal carcinoma (IDC) being the most common type. Lobular carcinoma (LC) accounts for approximately 10-14% of all BC [5]. Xiao and colleague’s review of 295,213 patients with a new diagnosis of invasive BC showed that the most common sites for distant metastases at the time of diagnosis were the bone (59.9%), lung (47.8%), liver (40.9%), and brain (38.8%) [2]. Metastasis to the gastrointestinal tract (GIT) is uncommon in BC, and most reported cases of GIT metastases were seen in patients with lobular subtype, with the ductal subtype metastasizing to the GIT less commonly [5].

McLemore’s retrospective review of 12,001 cases of metastatic BC between 1985 and 2000 identified 73 patients (0.6%) with a pathological affirmation of metastasis to the GIT and/or peritoneum. These were most frequently seen with the infiltrating lobular carcinoma (ILC) subtype, accounting for 44 out of 73 (60%) cases [3]. Only 24 out of 12,001 patients (0.2%) had colonic metastasis (CM). Montagna and colleagues’ review of 2,588 patients with BC found that 40 patients (1.55%) had GIT metastases, with only two having CM (0.07%) [6]. Both studies show that CM is extremely rare in BC. The mean interval between the diagnosis of BC to the diagnosis of GIT metastases was seven years [3], however, they did not report the mean interval time to the detection of CM, specifically.

Tasujimura and colleagues reported a case of a 51-year-old female presenting with abdominal pain due to an ascending colon mass and stenosis found on CT followed by colonoscopy [7]. Positron emission tomography-CT (PET-CT) showed an update in the ascending colon, mesentery, left breast, and left axilla, and a biopsy of the left breast confirmed ILC BC. She developed progressive abdominal pain and large bowel obstruction, requiring an emergency open ileocaecal resection, and the surgical specimen confirmed metastatic ILC in this region [7]. The patient recovered well and was discharged with ongoing treatment with letrozole.

Similarly, Noor and colleagues reported a case of a 68-year-old female with ILC BC, presenting 30 years after the initial diagnosis of BC, with symptoms of obstruction. CT confirmed circumferential narrowing and thickening in the distal sigmoid colon [8], and due to her history of BC, a decision was made to investigate with colonoscopy first. Colonoscopy revealed diffuse proliferation of neoplastic cells, with histochemistry confirmation of metastatic BC [8]. Further investigation with endoscopy also revealed gastric metastasis. No surgical resection was performed, and she continued to receive hormonal therapy and passed away four years later due to the progression of the disease [8].

The key difference between these reported cases and our presenting case is that our patient was asymptomatic, and the colonic metastasis was detected through a screening FOBT and random biopsies, as there were no suspicious lesions that prompted biopsy. No colonic lesions or enlarged lymph nodes were seen on recent surveillance CTs either. This may be due to ILC’s tendency to infiltrate the submucosal layer of GIT, which may appear as smooth bowel wall thickening on CT only, mimicking physiological peristalsis [9,10]. The mucosa may appear normal during colonoscopy, which suggests it would be falsely negative unless random biopsies are taken [10].

This case highlights that when assessing patients with a history of BC for positive FOBT, especially those with the ILC subtype, surgeons should be alerted to the possibility of colonic metastases; even if no malignant-appearing lesions were found on colonoscopy, random biopsies may be of use in detecting microscopic metastases, as ILC metastasis infiltrates the submucosa. A careful histological examination is also essential to correctly distinguish CM from a primary colonic malignancy, using specific stains for breast cancer, such as GATA-3 [3], as an estrogen receptor can be positive in primary colonic cancers in up to 70% of cases [11].

Correctly distinguishing a CM from a primary colonic malignancy is crucial as management would differ. There are no guidelines on the management of CM from BC, however, McLemore’s review of 12,001 BC patients compared the mean length of survival in those with GI metastasis and/or carcinomatosis between palliative surgery, systemic therapy, such as chemotherapy, and hormonal therapy, found that surgery was not shown to have a significant increase on overall median length of survival as compared to systemic therapy (28 months versus 26 months) [3]. In cases where BC and CM have not yet been confirmed, as in the case reported by Tasujimura [7], bowel resection may be performed. However, in patients with more advanced disease and multiple GI metastases and/or carcinomatosis, surgery should only be considered in the event of life-threatening complications, such as bowel obstruction with impending perforation, as surgery was not shown to have a survival benefit; they should be treated with systemic therapy [3].

## Conclusions

The combination of a long interval from the initial diagnosis of BC to the presentation and the rarity of isolated colonic metastases makes the diagnosis of colonic metastases difficult and easily missed. In addition to this, ILC metastasis to the GIT has been reported to infiltrate the submucosa first, which may give rise to falsely negative colonoscopy and CT scans when looking for metastatic lesions. Surgeons should carefully consider the possibility of CM when they are referred a patient with a history of ILC BC and a positive FOBT. There are no specific guidelines on how to manage CM from BC; however, surgical resection was not shown to improve the length of survival in patients with GI metastasis and/or carcinomatosis over chemotherapy and hormonal therapy and should only be considered in the event of life-threatening complications.
